# Supplementary material for: Fungal dual-domain LysM effectors undergo chitin-induced intermolecular, and not intramolecular, dimerization
Source: Plant Physiol. 2022 Aug 23;190(3):2033–44. doi: 10.1093/plphys/kiac391 (PMC9614474; doi:10.1093/plphys/kiac391)
Supplement: kiac391_Supplementary_Data [file kiac391_supplementary_data.pdf]

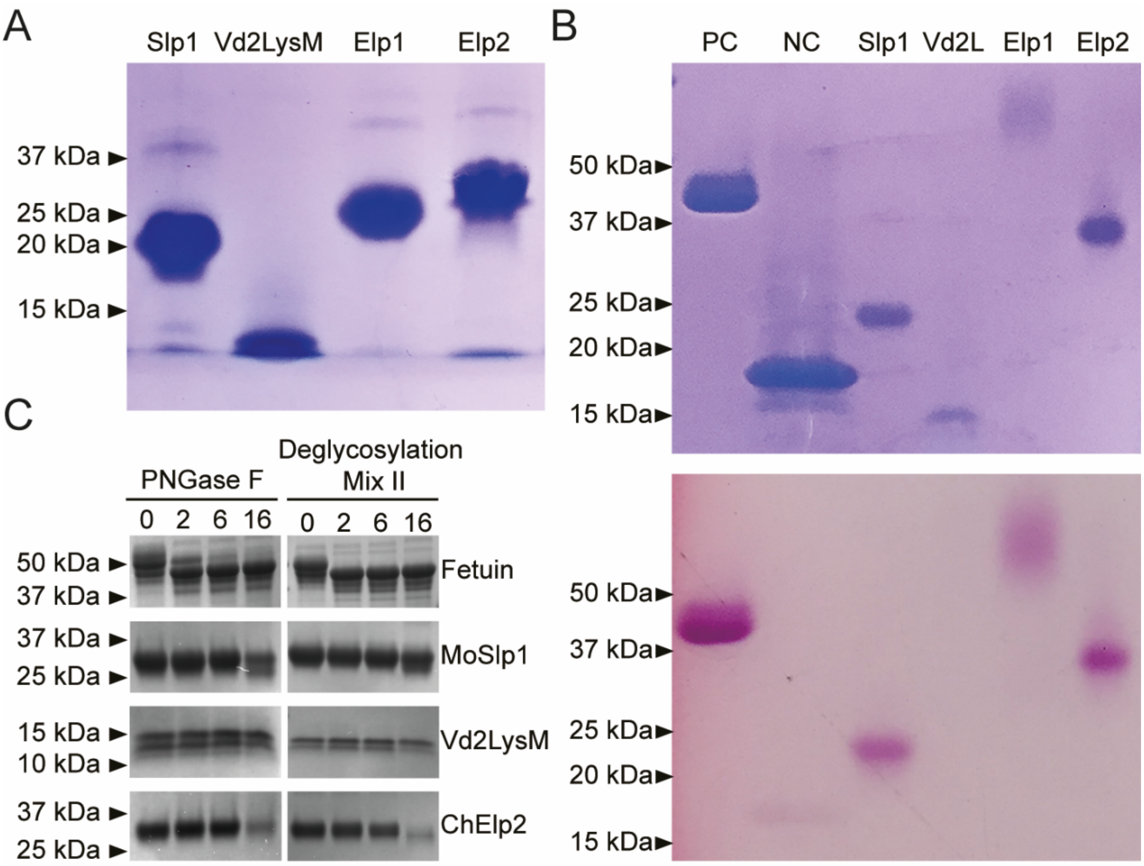

Supplemental Figure S1. Heterologous production of the four *P. pastoris*-produced dual-domain LysM effectors. (A) Protein polyacrylamide gel electrophoresis of 1 µL of the purified *Pichia pastoris*-produced dual LysM domain effectors *Magnaporthe oryzae* MoSlp1, *Verticillium dahliae* Vd2LysM, and *Colletotrichum higginsianum* ChElp1 and ChElp2 followed by Coomassie brilliant blue (CBB) staining. (B) CBB (upper panel) and glycoprotein staining (bottom panel) of polyacrylamide gels with Pichia pastoris-produced dual LysM domain effectors. PC and NC are positive (horseradish peroxidase) and negative (soybean trypsin inhibitor) control, respectively. (C) Deglycosylation assay performed with peptide:N-glycosidase F (PNGase F) and Protein Deglycosylation Mix II on MoSlp1, Vd2LysM, ChElp2 or fetuin as positive control. Protein samples were collected after different incubation times and subjected to polyacrylamide gel electrophoresis followed by CBB staining.

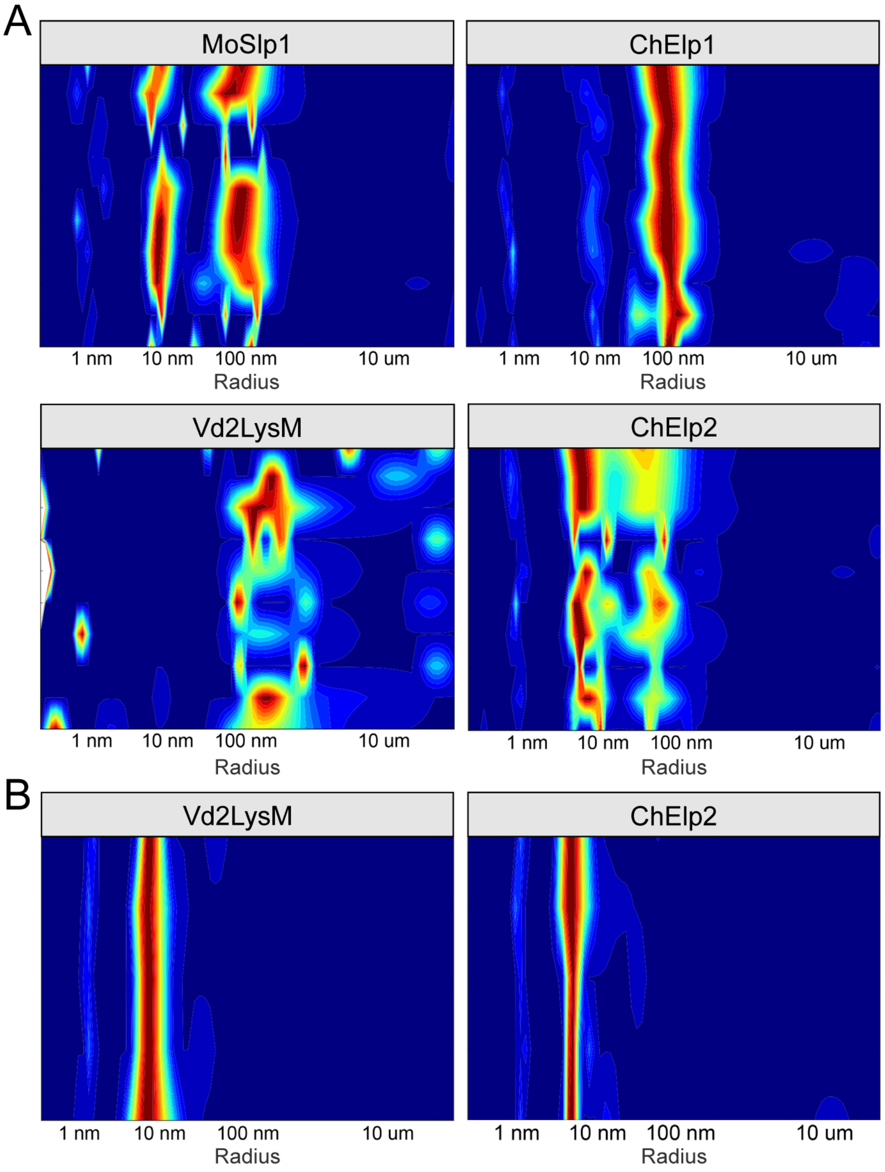

Supplemental Figure S2. Particle size distribution of dual-domain LysM effectors as determined by dynamic light scattering (DLS). The particle size distribution is shown as a colour scale heat map ranging from blue (lowest abundance) to red (highest abundance) for a particle size range of 1 nm to 100 μm. (A) Heat maps of the four *Pichia pastoris*-produced LysM effectors after initial purification and concentration. (B) Heat maps of Vd2LysM and ChElp2 after gel filtration and decyl β-D-maltopyranoside (DM) treatment.

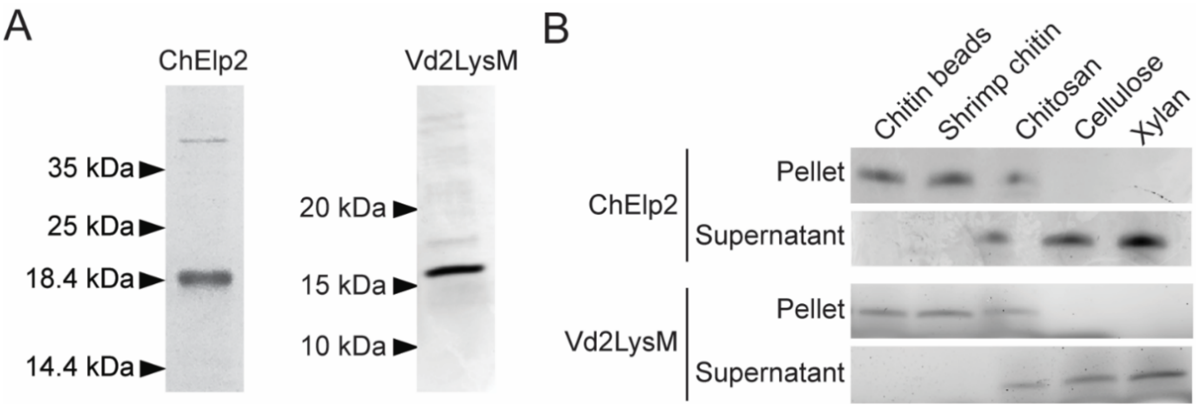

Supplemental Figure S3. *E. coli*-produced ChElp2 and Vd2LysM bind chitin. (A) ChElp2 and Vd2LysM were produced in *E. coli* and their quality was evaluated on protein polyacrylamide gel. (B) *E. coli*-produced ChElp2 or Vd2LysM were incubated with insoluble magnetic chitin beads, shrimp shell chitin, chitosan, cellulose and xylan for 6 hours. After centrifugation, pellet and supernatant fractions were analyzed on a protein polyacrylamide gel.

Supplemental Table S1. Primers used in this study.

Primers	Sequences
**His-Flag-MoSlp1-F**	CGGTATGAATTC**ATG**CATCATCATCATCATCAT CCCGACTACAAGGACGACGATGACAAGGCCATGCCTCAGGCAAC
**His-Flag-MoSlp1-R**	CGGTATGCGGCCGCCTAGTTCTTGCAGATGGGGATGTTG
**His-Flag-Vd2L-F**	CGGTATGAATTC**ATG**CATCATCATCATCATCAT CCCGACTACAAGGACGACGATGACAAGTACCGAAGGACATGCAGTCATACC
**His-Flag-Vd2L-R**	CGGTATGCGGCCGCTTAGTTCCAGCTGCACGGC
**His-Flag-ChElp1-F**	CGGTATGAATTC**ATG**CATCATCATCATCATCAT CCCGACTACAAGGACGACGATGACAAGCTCCCTCAGGCTACCCCGACCACG
**His-Flag-ChElp1-R**	CGGTATGCGGCCGCTTAACAGACGGGGATGTTGATGACATCGC
**His-Flag-ChElp2-F**	CGGTATGAATTC**ATG**CATCATCATCATCATCAT CCCGACTACAAGGACGACGATGACAAGCTCCCTGTCTGTGACGCCACTAC
**His-Flag-ChElp2-R**	CGGTATGCGGCCGCCTAACACACAGGGACGTTGATGACC
**MoSlp1-F**	CGGTATGAATTCGCCATGCCTCAGGCAAC
**MoSlp1-R**	CGGTATGCGGCCGC**TTATTA**CTAGTTCTTGCAGATGGGGATGTTG
**Vd2L-F**	CGGTATGAATTCTACCGAAGGACATGCAGTCATACC
**Vd2L-R**	CGGTATGCGGCCGC**TTATTA**TTAGTTCCAGCTGCACGGC
**ChElp1-F**	CGGTATGAATTCCTCCCTCAGGCTACCCCGACCACG
**ChElp1-R**	CGGTATGCGGCCGC**TTATTA**TTAACAGACGGGGATGTTGATGACATCGC
**ChElp2-F**	CGGTATGAATTCCTCCCTGTCTGTGACGCCACTAC
**ChElp2-R**	CGGTATGCGGCCGC**TTATTA**CTAACACACAGGGACGTTGATGACC
**FLAG-ChElp2-F**	CGGTATGAATTCCCCGACTACAAGGACGACGATGACAAGCTCCCTGTCTGTGACGCC ACTAC

*The coding sequence for the 6×His-tag is indicated with cyan font, the coding sequence for the FLAG-tag is indicated with blue font, and restriction enzyme recognition sites are black underlined.

Supplemental Table S2. Constructs used in this study for protein production.

Construct name	Primers used	Restriction sites	Destination vector
pPIC9-MoSlp1	His-Flag-MoSlp1-F<br>His-Flag-MoSlp1-R	*Eco*RI<br>*Not*I	pPIC9
pPIC9-Vd2LysM	His-Flag-Vd2L-F<br>His-Flag-Vd2L-R	*Eco*RI<br>*Not*I	pPIC9
pPIC9-ChElp1	His-Flag-ChElp1-F<br>His-Flag-ChElp1-R	*Eco*RI<br>*Not*I	pPIC9
pPIC9-ChElp2	His-Flag-ChElp2-F<br>His-Flag-ChElp2-R	*Eco*RI<br>*Not*I	pPIC9
pETSUMO-MoSlp1	MoSlp1-F<br>MoSlp1-R	*Eco*RI<br>*Not*I	pETSUMO
pETSUMO-Vd2LysM	Vd2L-F<br>Vd2L-R	*Eco*RI<br>*Not*I	pETSUMO
pETSUMO-ChElp1	ChElp1-F<br>ChElp1-R	*Eco*RI<br>*Not*I	pETSUMO
pETSUMO-ChElp2	ChElp2-F<br>ChElp2-R	*Eco*RI<br>*Not*I	pETSUMO
pETSUMO-FLAG-ChElp2	FLAG-ChElp2-F<br>ChElp2-R	*Eco*RI<br>*Not*I	pETSUMO

Supplemental Table S3. Final concentrations of the four *P. pastoris*-produced LysM effectors.

Protein	Concentration
**MoSlp1**	10 mg/ml
**Vd2LysM**	7.35 mg/ml
**ChElp1**	9.0 mg/ml
**ChElp2**	14 mg/ml

Supplemental Table S4. Lysis buffer composition (20 mL).

Amount	Reagent	Company
**18 mL**	50 mM Tris, 150 nM NaCl, pH 8.5	
**2 mL**	Glycerol	VWR, Ohio, USA
**120 mg**	Lysozyme from chicken egg white	Sigma-Aldrich, Missouri, USA
**80 mg**	Sodium deoxycholate	Sigma-Aldrich, Missouri, USA
**1.25 mg**	Deoxyribonuclease I	Sigma-Aldrich, Missouri, USA
**1 pill**	Protease inhibitor cocktail	Roche, Mannheim, Germany
